# Comparison of ankle and subtalar joint complex range of motion during barefoot walking and walking in Masai Barefoot Technology sandals

**DOI:** 10.1186/1757-1146-4-1

**Published:** 2011-01-02

**Authors:** Sophie Roberts, Ivan Birch, Simon Otter

**Affiliations:** 1 Outpatients Department, Parkside Hospital, 53 Parkside, London, SW19 5NX, UK; 2 Thames Valley University, Paragon House, Boston Manor Road, Brentford, Middlesex, TW8 9GA, UK; 3 University of Brighton, School of Health Sciences, 49 Darley Rd, Eastbourne, BN20 7UR, UK

## Abstract

**Background:**

Masai Barefoot Technology (MBT, Switzerland) produce footwear which they claim simulate walking barefoot on soft undulating ground. This paper reports an investigation into the effect of MBT sandals on the motion of the ankle and subtalar joint complex during walking.

**Methods:**

Range of motion data was collected in the sagittal, frontal and transverse plane from the ankle and subtalar joint complex from 32 asymptomatic subjects using the CODA MPX30 motion analysis system during both barefoot walking and walking in the MBT sandal. Shod and un-shod data were compared using the Wilcoxon signed ranks test.

**Results:**

A significantly greater range of motion in the frontal and sagittal planes was recorded when walking in the MBT sandal (p = 0.031, and p = 0.015 respectively). In the transverse plane, no significant difference was found (p = 0.470).

**Conclusions:**

MBT sandals increase the range of motion of the ankle and subtalar joint complex in the frontal and sagittal planes. MBT footwear could therefore have a role to play in the management of musculoskeletal disorders where an increase in frontal and sagittal plane range of motion is desirable.

## Background

With the growth in the health and fitness industry, sports footwear has shown technological advances and diversification. The introduction of footwear such as the MBT shoe (manufactured by Masai Barefoot Technologies) is an example of this diversification. MBT have based their shoe design on observations of the Masai, a semi-nomadic tribe from Africa who are well known for their posture and for walking long distances on uneven terrain. The MBT shoe construction is based on a mid-sole pivot with a rounded sole in the anterior-posterior direction, and a soft heel pad. It is claimed that the effect of the angled soft sole not only creates anterior/posterior facilitation of movement, by purposefully creating medial and lateral instability in the shoe [1].

According to Romkes et al [2], MBT shoes have been used to treat foot problems such as hallux valgus, pes planus, heel and tendo-achilles pain as well as circulatory problems. Nigg et al [3] found that MBT shoes may be useful as a training device for stability and muscle strengthening adding another dimension to their use. However, New et al [4] and Nigg et al [3] found that subjects from many of the studies conducted using MBT shoes received an instruction session in which they were taught how to use the shoes correctly by a qualified instructor and given a long training period to acclimatise to the shoes. As such there remains debate as to whether the reported changes in gait were as a result of the MBT shoe or the professional instruction in gait and posture the subjects received and the training period they had [4].

Nigg et al [3] described that an increase in the angle of ankle dorsiflexion in MBT shoes was evident compared to a standard training shoe. Vernon et al [5] also reported subjects exhibited significantly higher maximum dorsiflexion angle at the ankle joint when wearing MBT shoes. However, it should be noted that in both these studies, the markers for the motion analysis system used to detect movement were placed on the outer surface of the shoe and therefore their ability to indicate ankle dorsiflexion could be disputed. Therefore, the scientific evidence for the clinical use of the MBT shoe has yet to be presented in the literature, in particular the changes in function of the ankle and subtalar joint complex between barefoot walking and walking in the MBT shoe claimed by the manufacturers. This study was designed to establish whether there was any affect of the MBT shoe on human locomotion, in particular to identify what differences there are between the range of motion of the ankle and subtalar joint complex of the foot during walking in an MBT shoe compared to barefoot walking.

## Methods

### Participants

A total of 32 healthy subjects between the ages of 18 and 35 were recruited and provided with informed written consent to participate in the study from the staff/student population at the University of Brighton. The criteria for inclusion and exclusion used in the subject selection process are shown in Table 1. Ethical approval was granted from the University Of Brighton School Of Health Professions Ethics Committee. Data were collected in the School Of Health Professions Human Movement Laboratory using the CODA mpx30 active marker motion analysis system (Charnwood Dynamics, Leicestershire, UK).

**Table 1:** Inclusion and exclusion criteria

Inclusion	Female participants must fall within shoe size 4 - 7
	Male participants must fall within shoe size 7 - 10
	18 and 35 years old
	Healthy and injury-free
Exclusion	Pain or dysfunction in the lower limb or have done for the last 6 months, which may affect their ability to ambulate
	Previous use of an MBT shoe

### Data collection

**Figure 1:**
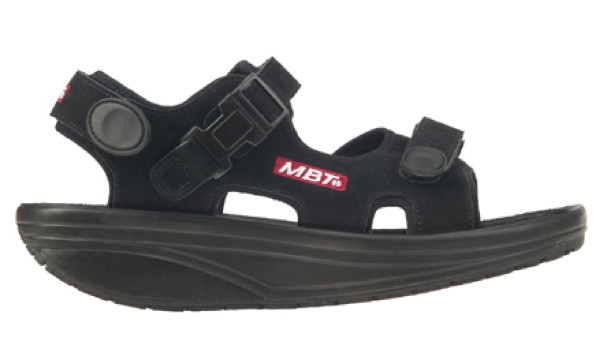
**MBT sandal used in the study**.

The first set of data was collected with participants walking barefoot. The CODA measurement framework was aligned so that Y-axis was anterior/posterior, the X-axis was medial/lateral and the Z -axis was vertical. The marker placement model used for the current study was based on the model reported by Birch and Deschamp [6]. To enhance the anatomical reliability of marker placement, an MBT sandal (see Figure 1) was used for data collection so the active markers for the CODA system could be placed directly on the anatomical landmarks of each subject and not estimated on the outside of the shoe. According to the MBT manufacturers the unique strap system of the sandal cradles the whole foot providing the same function as the MBT shoe [1]. The appropriate size MBT sandal was selected for each subject using the MBT shoe fit guidelines and the subjects were invited to wear these for 20 minutes to familiarize themselves with the sandal. The CODA sensors were placed on the anatomical landmarks detailed in Table 2. Motion data were collected from each subject while walking on a 10-metre walkway with the CODA sensor unit positioned at each end. A 10 Hz filter was applied to all data.

**Table 2:** Anatomical landmarks for the CODA marker placement

Marker 1	Fibular head
Marker 2	Lateral malleolus

Marker 3	Medial malleolus

Marker 4	Lateral calcaneus

Marker 5	Mid calcaneus

Marker 6	Medial calcaneus

### Statistical analysis

A single mid-gait footstep was selected from each data set; only the right foot was studied owing to the complexity of the marker placement model used. A co-ordinate transform was applied using the CODA software. The fibula head, medial and lateral malleolus markers were used to determine the frontal, sagittal and transverse motion from which calcaneal motion could be measured. Calcaneal motion data measured relative to the leg was exported into Microsoft Excel (Microsoft Corporation, Redmond, WA, USA). These data were then cleaned for outliers and the range of motion was calculated for each subject by subtracting the minimum angle recorded from the maximum angle. Data were analysed using SPSS version 15 (SPSS Inc Chicago, IL, USA). Shod and un-shod data were compared using the combination of graphs, descriptive statistics and the Wilcoxon signed ranks test for statistical significance.

## Results

**Figure 2:**
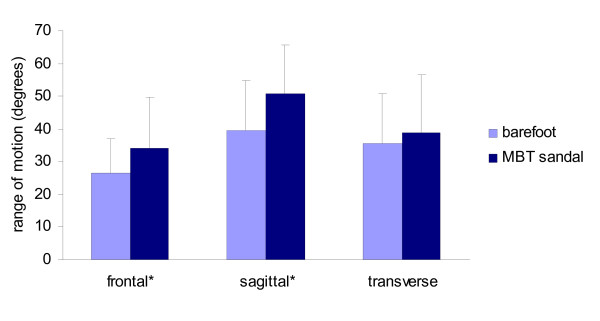
**Mean (± SD) ranges of motion when walking barefoot and in the MBT sandal**. *p < 0.05.

The range of motion was calculated for the subtalar and ankle joint complex in the frontal, sagittal and transverse planes during one footstep using the CODA MPX30 (Figure 2). Overall, there was an increase in the range of motion in all three planes of motion from walking barefoot to walking in an MBT sandal. The range increased more in some planes than others, with the greatest increase in the sagittal plane. However, a minority of subjects clearly demonstrated a decrease in range of motion. Mean, standard deviation and range of data are illustrated in Table 3 with an increase in the mean values in range of motion from walking barefoot and walking in the MBT shoes in all three planes of motion being noted. Larger standard deviations were noted in the MBT sandals in the frontal plane compared to the two other planes.

**Table 3:** Descriptive statistics for the range of motion differences between barefoot and MBT walking trials

Plane	Condition	Mean	SD	Minimum	Maximum
Frontal	Barefoot	26.3	10.6	5.5	45.5
	
	MBT sandal	34.0	15.6	10.9	66.3

Sagittal	Barefoot	39.4	15.5	15.1	69.6
	
	MBT sandal	50.6	15.1	22.1	84.0

Transverse	Barefoot	35.7	15.1	8.1	58.0
	
	MBT sandal	38.8	17.7	7.0	67.3

The range of frontal and sagittal plane motion was significantly higher when wearing MBT sandal compared to walking barefoot (frontal p = 0.031, sagittal p = 0.015). However, for the transverse plane, although the mean range of motion was higher when wearing the MBT sandal than when walking barefoot; this difference was not statistically significant (p = 0.470).

## Discussion

The increase in sagittal range of motion on walking in the MBT sandal in our study could be attributed to the rounded sole design of the sole of the MBT footwear. This sole design may lead to increased dorsiflexion of the ankle and subtalar joint complex at initial contact followed by an increase in plantarflexion during the propulsive phase due to the rounded anterior edge of the sole. Movement of the ankle and subtalar joint movement during stance phase would be encouraged by the rounded sole creating inertia. This could perhaps increase range of motion through the ankle and subtalar joint complex in the sagittal plane compared to walking barefoot on a flat surface and would possibly explain the results found in this study.

Furthermore, the design of the MBT shoe creates an uneven surface for the foot by using low density materials as part of the sole construction. Therefore, an increase not only in the sagittal plane movement but also frontal plane movement of the ankle and subtalar joint could be expected, and may explain our findings of a statistically significant increase in the range of motion in the frontal plane in the MBT sandal compared to walking barefoot. In this study, only the total range of frontal plane motion was measured, rather than the amount of inversion and eversion. According to Nigg et al [3], the rotational inversion loading was higher in an MBT shoe compared to a standard training shoe, suggesting that the increased range of frontal plane movement found in this current study may primarily be in the direction of inversion.

The results of the study demonstrated that although there was a small increase in the range of motion in the transverse plane, this difference was not statistically significant. According to Nester et al [7], although the ankle is often considered to have little capacity to move in the transverse plane, it is capable of considerable movement in the transverse plane (greater than 15 degrees). In our study, transverse plane range of motion varied between 7 and 57 degrees. In terms of differences between individuals, our data indicated that some subjects demonstrated a decrease in the range of motion when walking in the MBT sandal. This could have resulted from the subjects having insufficient time to get used to the shoes. Equally, some subjects reported feeling highly unstable in the shoes, which may have caused their ankle and subtalar joints to function with a higher range of motion compared to walking barefoot.

There are conflicting views on the indications for MBT footwear in the medical community and guidelines for clinicians need to be implemented so that the footwear can be appropriately prescribed. The observed increase in the range of motion at the ankle and subtalar joint complex in the MBT sandal could potentially be beneficial in certain patient groups, particularly in those where the decreased range of motion in these joints are putting strain on other parts of the musculoskeletal kinetic chain. For example, according to Monaghan et al [8], the therapeutic goal for chronic ankle instability is to re-train muscles to improve control during gait, and an MBT sandal may be an appropriate tool for this. However, if walking in an MBT sandal demands greater subtalar and ankle joint range of motion than is available, soft tissue damage may occur. Further research in specific patient groups needs to be conducted to aid appropriate prescription of the MBT sandal.

## Conclusions

MBT sandals produce a statistically significant increase in frontal and sagittal plane ranges of motion of the subtalar and ankle joint complex during gait. However, transverse plane motion was not significantly altered. MBT footwear could therefore have a role to play in the management of musculoskeletal disorders where an increase in frontal and sagittal plane range of motion is considered desirable.

## Competing interests

The authors declare that they have no competing interests.

## Authors' contributions

All authors have made substantial contributions to the conception and design of the study, acquisition of data, analysis and interpretation of data, drafting the article and revising it critically for important intellectual content, and final approval of the version to be submitted. SR carried out the study design, data collection and write up. IB: assisted in study design, and data collection/analysis. SO: assisted in data analysis, particularly the statistical analysis and also the discussion.

## References

[B1] Masai Barefoot Technology http://www.mbt.com

[B2] Romkes J Rudmann C Brunner R Changes in gait and EMG when walking with the Masai Barefoot Technique Clin Biomech 2006 21 75 81 10.1016/j.clinbiomech.2005.08.003 16169641

[B3] Nigg B Hintzen S Ferber R The effect of an unstable shoe construction on lower extremity gait characteristics Clin Biomech 2006 21 82 88 10.1016/j.clinbiomech.2005.08.013 16209901

[B4] New P Pearce J The effects of Masai Barefoot Technology footwear on posture: an experimental designed study (dissertation) 2007 University of Southampton School of Health Professions

[B5] Vernon T Wheat J Naik R Pettit G Changes in gait characteristics of a normal, healthy population due to an unstable shoe construction (dissertation) 2004 Sheffield Hallam University Centre for Sports and Exercise

[B6] Birch I Deschamps K The in vitro reliability of the CODA MPX30 as the basis for a method of assessing the in vivo motion of the subtalar joint J Am Podiatr Med Assoc 2010 in press 10.7547/1010400 21957271

[B7] Nester C Van De Linden M Bowker P Effect of foot orthoses on the kinematics and kinetics of normal walking gait Gait Posture 2003 17 180 187 10.1016/S0966-6362(02)00065-6 12633779

[B8] Monaghan K Delahunt E Caulfield B Increasing the number of gait trial recordings maximises intra-rater reliability of the coda motion analysis system Gait Posture 2007 25 303 315 10.1016/j.gaitpost.2006.04.011 16730177

